# Appetite regulation is independent of the changes in ghrelin levels in pregnant rats fed low-protein diet

**DOI:** 10.14814/phy2.12368

**Published:** 2015-04-23

**Authors:** Haijun Gao, Daren T Tanchico, Uma Yallampalli, Meena P Balakrishnan, Chandra Yallampalli

**Affiliations:** 1 Department of Obstetrics & Gynecology, Baylor College of MedicineHouston, Texas; 2 Texas Children's HospitalHouston, Texas

**Keywords:** Diet intake, ghrelin, low-protein diet, pregnancy, rat

## Abstract

Gestational protein restriction causes hypertension in the adult offspring. Very little is known about the food intake regulation and ghrelin signaling in pregnant dams fed a low-protein (LP) diet. We hypothesized that diet intake and ghrelin signaling are altered in pregnant rats fed the low-protein diet. Sprague–Dawley rats were fed a control (CT) or LP diet from Day 3 of pregnancy. Diet intake and body weight were monitored daily. Expression of ghrelin production-related genes in the stomach and appetite-related genes in the hypothalamus was analyzed by real-time PCR. Plasma levels of total and active ghrelin, growth hormone and leptin were measured by ELISA. Main results include: (1) Daily diet intake was greater in the LP group than in the CT group in early pregnancy, but substantially lower in late pregnancy; (2) Daily gain in body weight was substantially lower in the LP group in late pregnancy; (3) Expression of ghrelin production-related genes in the stomach and plasma total ghrelin levels were increased in LP group in late pregnancy; (4) Plasma active ghrelin levels were elevated in the LP group at mid-late pregnancy, but growth hormone and leptin levels were uncorrelated with active ghrelin in late pregnancy; and (5) Hypothalamic expression of ghrelin-stimulated genes in LP rats was unassociated with the changes in both plasma ghrelin levels and the diet intake. Taken together, the appetite in LP rats is greater in early pregnancy but reduced at late pregnancy, possibly due to ghrelin insensitivity in appetite regulation.

## Introduction

Studies on fetal programming of cardiovascular and metabolic diseases have enormous clinical significance, and various animal models have used nutritional (Goyal et al. 2009, 2010), hormonal (Dodic et al. 2001; Forhead and Fowden 2004; Connors et al. 2010), and surgical (Alexander 2003) manipulations during gestation to understand the mechanisms involved. Among these models, pregnant rats given a low-protein diet have been widely used (Kautzky-Willer and Handisurya 2009). Low-protein diets during pregnancy decrease both placental and fetal weights (Jansson et al. 2006; Gao et al. 2012a,b), and both male and female offspring develop hypertension in adulthood (Langley-Evans et al. 1996, 1999; Gangula et al. 2005; McMullen and Langley-Evans 2005a,b; Sathishkumar et al. 2012). Although it is well established that nutrients including protein and amino acids are critical for fetal growth, to date, there are few studies investigating systematically the diet intake and body weight gain in pregnant rats fed a low-protein diet (Desai et al. 1996; Fernandez-Twinn et al. 2003).

Appetite is regulated by interaction between the central nervous system and peripheral organs. Certain regions in the hypothalamus, including in particular the arcuate nucleus (ARC), integrate various signals such as ghrelin, leptin, and insulin in regulating appetite (Naslund and Hellstrom 2007; Ladyman et al. 2010; Sobrino et al. 2014). Among the known appetite-regulating signals, ghrelin is the only peripherally produced hormone which is known to stimulate appetite and thus, is considered to be a critical regulator of appetite and nutritional status (Fernandez-Fernandez et al. 2006; Angelidis et al. 2012).

Ghrelin is secreted by the stomach in response to hunger and fasting (Toshinai et al. 2001; Cummings et al. 2004). In the stomach, the ghrelin precursor (prepro-ghrelin) is cleaved into proghrelin, which is further cleaved to produce ghrelin, a 28-amino acid peptide termed desacyl ghrelin; desacyl ghrelin is acetylated by the enzyme MBOAT4 [membrane bound O-acyltransferase domain containing 4, also known as GOAT (ghrelin O-acyltransferase)] (Gutierrez et al. 2008; Yang et al. 2008) and becomes ‘active ghrelin’ with a high affinity to GHSR1a (growth hormone secretagogue type 1a receptor) (Kojima et al. 1999). Active ghrelin is inactivated by the enzyme BCHE (butyrylcholinesterase) (De et al. 2004).

Active ghrelin produced in the stomach is released into the blood circulation, and it stimulates appetite in part by its actions on NPY/AgRP (neuropeptide Y/Agouti-related peptide) expressing neurons in the ARC (Kojima and Kangawa 2010). Briefly, active ghrelin binds to GHSR1a and stimulates phosphorylation of AMPK (AMP-activated protein kinase). AMPK phosphorylates ACC (acetyl-CoA carboxylase) which promotes fatty acid metabolism and CPT1 (carnitine palmitoyltransferase) activation. Free radical production in fatty acid metabolism in mitochondria stimulates expression of *Ucp2* (uncoupling protein 2) (Andrews et al. 2008), which is followed by increased diet intake. In addition, NPY/AgRP hormones suppress the activity of CART/POMC (cocaine-amphetamine regulated transcript/proopiomelanocortin) expressing neurons in the ARC (Funahashi et al. 2003; Naslund and Hellstrom 2007). In addition, active ghrelin induces release of growth hormone from the pituitary (Kojima and Kangawa 2005), which is initiated by active ghrelin binding to GHSR1a in growth hormone-releasing hormone cells in the ARC (Sun et al. 2004).

To date, little is known about ghrelin-induced appetite stimulation during pregnancy, and specifically in pregnant dams fed a low-protein diet. During pregnancy, increased appetite and food intake are critical for facilitating energy storage for the high metabolic demands of pregnancy and subsequent lactation (Ladyman et al. 2010). It has been reported that ghrelin production is stimulated by dietary protein, and reduced by fat and carbohydrates (Erdmann et al. 2003), thus the reduced protein content in the LP diet may affect ghrelin-regulated diet intake in pregnant dams. To test this, we investigated, in pregnant rats: (1) dietary intake during gestation; (2) daily gain in body weight; (3) plasma levels of total and active ghrelin; (4) expression of ghrelin production-related genes *Ghrl*, *Mboat4,* and *Bche* in the stomach; and (5) expression of appetite stimulation marker genes (*Npy*, *Agrp*), appetite inhibition marker genes (*Pomc*, *Cart*) and ghrelin signaling pathway-related genes (*Gshr*, *Fasn*, *Cpt1a*, *Cpt1c*, *Ucp2*) in the whole hypothalamus as well as in the ARC.

## Materials and Methods

### Animal and diets

All procedures were approved by the Animal Care and Use Committee at Baylor College of Medicine and were in accordance with those published by the US National Institutes of Health Guide for the Care and Use of Laboratory Animals (2011). Timed pregnant Sprague–Dawley rats weighing between 175 and 225 g were purchased from Harlan (Houston, TX). At Day 3 of pregnancy (Day 1 was determined by the presence of sperm in the virginal smear after breeding), rats were randomly divided into two dietary groups, housed individually and fed a control (CT, 20% casein) or low-protein (LP, 6% casein) diet until they were killed on Days 10, 14, 18, 19, 21, or 22 of pregnancy (*n* = 8–10 rats/diet/day of pregnancy). Both diet intake and body weights were recorded at 24-h intervals, and assessed daily between 7.00AM and 8.00AM. The isocaloric low-protein and normal-protein diets were purchased from Harlan Teklad (Cat. TD.90016 and TD.91352, respectively; Madison, WI). The contents of these diets were described in a previous study and isocaloric value of the diet was maintained by the increased sucrose content (Vehaskari et al. 2001). The contents of other nutrients except methionine are also similar between the LP diet and normal CT diet. The content of methionine was reduced in the LP diet proportionately. In pregnant rats fed with the LP diet, plasma levels of total nonessential amino acids were increased and total essential amino acids were reduced, whereas no changes were observed in total free amino acids (Gao et al. 2012a). In contrast, in fetal plasma all amino acids except threonine were unchanged in late pregnancy (Rees et al. 1999). In addition, our previous studies showed that the LP diet did not affect the litter size or delivery time (Gangula et al. 2005; Jansson et al. 2006), but reduced both placental and fetal weights (Gao et al. 2012a,b) and neonatal survival rate (Gangula et al. 2005). All offspring were hypertensive as they became adults with more severe and earlier onset in males (3-month old), compared to females (6-month old) (Gangula et al. 2005; Sathishkumar et al. 2012).

### Tissue collection

Rats were killed by CO_2_ inhalation between 8.00 am and 10.00 am on Days 10, 14, 18, 19, 21, or 22 of pregnancy. Whole blood was collected by left ventricle puncture using a 10 mL syringe and an 18G needle and injected into BD Vacutainer blood collection tube containing K2-EDTA (Cat. 36643, BD, Franklin Lakes, NJ). The blood was then mixed with protease inhibitor, Pefabloc SC (Cat. 76307; Sigma-Aldrich, St. Louis, MO) to a final concentration of 1 mg/mL and centrifuged at 3000 *g* for 15 min at 4°C. The supernatant was aliquotted, snap frozen in liquid nitrogen and stored at −80°C until analysis.

The stomach was trimmed free of fat and pancreatic tissues, slit open and the contents were removed by wiping with Kimwipe tissues. The cleaned stomach was snap frozen in liquid nitrogen and stored at −80°C until analysis.

The hypothalamus and ARC were collected using the dissection procedures described in a previous report (Heffner and Seiden 1980). Briefly, the brain was quickly removed and oriented properly in the precooled rat brain matrix (coronal sections; Cat. RBM-4000C; ASI Instruments, Warren, MI). Square blades were placed into the given positions in brain matrix according to a rat brain atlas (Paxinos and Watson 1998). To dissect the hypothalamus, blades were placed at the middle of optic chiasm and the end of the hypothalamus (3.5–8 mm; Interaural), and the thalamus was trimmed off by cutting longitudinally at the tip of Corpus Callosum. To dissect the ARC region, blades were placed at 2 and 5 mm back from the middle of optic chiasm (6.88–4.48 mm; Interaural). The section was trimmed to the thickness of 3 mm from the caudal surface of the hypothalamus (−2.12 to −4.52 mm; Bregma). The sections of the hypothalamus and ARC were snap frozen in liquid nitrogen and stored at −80°C until analysis.

### RNA extraction and RT-PCR

Total RNA was extracted from the stomach, the hypothalamus, and ARC (*n* = 8–10 rats per diet per day of pregnancy) by Trizol reagent (Cat. 15596-018; Invitrogen, Carlsbad, CA) according to the manufacturer's protocol. The possible genomic DNA in total RNAs was digested with RNA free DNase I (Cat. 79254; Qiagen Inc., Valencia, CA), followed by clean-up procedures using a Qiagen RNeasy Minikit (Cat. 74104; Qiagen). In all these procedures the manufacturer's instructions were followed. Complementary DNA (cDNA) was synthesized from 1 *μ*g of total RNA by reverse transcription in a total volume of 20 *μ*L by using a MyCycler Thermal Cycler (Cat. 170-9703, Bio-Rad Laboratories, Hercules, CA) under the following conditions: One cycle at 28°C for 15 min, 42°C for 50 min, and 95°C for 5 min.

### Quantitative real-time PCR

Real-time PCR detection was performed on a CFX96 Real Time PCR Detection System (Cat. 184-5096; Bio-Rad). Primers were designed using Primer 3 Version 4 and are shown in Table1. Syber Green Supermix (Cat. 170-8882; Bio-Rad) was used for amplification of *Ghrl*, *Mboat4,* and *Bche* (ghrelin production-related genes) in the stomach, *Npy* and *Agrp* (marker genes for appetite stimulation), *Pomc* and *Cart* (marker genes for appetite inhibition), *and Gshr, Ucp2*, *Fasn*, *Cpt1a,* and *Cpt1c* (ghrelin signaling-related genes) in the hypothalamus, and *Npy* and *Agrp*, *Pomc* and *Cart* in the ARC region of the hypothalamus. *Actb* served as internal control to normalize target gene expressions in these organ/tissues. The reaction mixture was incubated at 95°C for 10 min and cycled according to the following parameters: 95°C for 30 sec and 60°C for 1 min for 40 cycles. Amplification of a single product was confirmed by melting curve analysis. A negative control without cDNA was performed to test primer specificity. The relative gene expression was calculated by use of the threshold cycle (C_T_) *Actb*/C_T_ target gene.

**Table 1:** Quantitative real-time PCR primers.

Gene	Forward primer (5′→3′)	Reverse primer (5′→3′)	GenBank accession No.	Product size (bp)
*Ghrl*	AGCCCAGCAGAGAAAGGAAT	GTGGCTGCAGTTTAGCTGGT	NM_021669.2	50
*Mboat4*	CCAGGAGCAGGAGTTCTTTG	AGTGCAGGGAAAAAGAGCAA	NM_001107317.2	79
*Bche*	AGTGGGCGTTAACAAGGATG	AAACCAGGAGCACCGTACAC	NM_022942.1	57
*Npy*	TACTCCGCTCTGCGACACTA	TGTCTCAGGGCTGGATCTCT	NM_012614.2	72
*Agrp*	AAGCTTTGGCAGAGGTGCTA	GACTCGTGCAGCCTTACACA	NM_033650.1	76
*Cartpt*	CCCTACTGCTGCTGCTACCT	CACGGCAGAGTAGATGTCCA	NM_017110.1	89
*Pomc*	GCTTCATGACCTCCGAGAAG	TCTTGATGATGGCGTTCTTG	NM_139326.2	66
*Ghsr*	TCCACGTGGGAAGATACCTC	CAGGTTGCAGTACTGGCTGA	NM_032075.3	83
*Fasn*	GGCATCATTGGGCACTCCTT	GCTGCAAGCACAGCCTCTCT	NM_017332.1	83
*Cpt1a*	CAGCTCGCACATTACAAGGA	TGCACAAAGTTGCAGGACTC	NM_031559.2	124
*Cpt1c*	CACACCTGTTCGATGTCCAC	TTGATTGCTTGCTGGAGATG	NM_001034925.2	144

### ELISA on plasma total and active ghrelin

Total ghrelin in plasma from pregnant rats was measured by Enzyme-Linked Immuno Sorbent Assay (ELISA) kit (Cat. EZRGRT-91K; Millipore, Billerica MA), according to the manufacturer's instructions. Per manufacturer, the lowest detectable level of total ghrelin is 0.04 ng/mL; the specificity for active and des-octanoyl ghrelin is 85% and 100%, respectively; the intraassay CV is 2.0–3.2%; the interassay CV is 1.1–1.7%.

Active ghrelin in plasma from pregnant rats was measured by ELISA kit (Cat. EZRGRA-90K; Millipore), according to the manufacturer's instructions. Per manufacturer, the lowest detectable level of rat active ghrelin is 8 pg/mL; the specificity for active ghrelin is 100%; the intraassay CV is 1.0–6.0%; the interassay CV is 1.0–5.0%.

### ELISA on plasma growth hormone

Growth hormone in plasma from pregnant rats was measured by ELISA kit (Cat. KRC5311, Life Technologies, Grand Island, NY), according to the manufacturer's instructions. Per manufacturer, the lowest detectable level of rat growth hormone is 0.5 ng/mL; the specificity for growth hormone is 100%; the intraassay CV is 3.3–6.1%; the interassay CV is 3.1–6.0%.

### ELISA on plasma leptin

Leptin in plasma from pregnant rats was measured by ELISA kit (Cat. EZRL-83K; Millipore, Billerica MA), according to the manufacturer's instructions. In this assay, the lowest detectable level of rat leptin is 0.08 ng/mL; the specificity for leptin is 100%; the intraassay CV is 1.9–2.5%; the interassay CV is 3.0–3.9%.

### Statistical analysis

All quantitative data were subjected to least-squares analysis of variance (ANOVA) using the general linear models procedures of the Statistical Analysis System (Version 9.3, SAS Institute, Cary, NC). Daily diet intake was presented as the ratio of diet intake (gram) to the body weight (gram) measured at the time of diet given to rats every morning. Daily gain in body weight was presented as the difference in body weight between a given day and 1 day before. Accumulative gain in body weight was presented as the difference in body weight between a given day and Day 3 of pregnancy. Data on daily diet intake, daily gain in body weight, accumulative gain in body weight, gene expression, and plasma hormone levels were analyzed for effects of day of pregnancy, diet treatment, and their interaction. Log transformation of variables was performed when the variance of data were not homogenous among treatment groups, as assessed by the Levene's test. A *P*-value ≤ 0.05 was considered significant; a *P*-value > 0.05 and ≤0.10 was considered a trend toward significance. Data were presented as least-squares means (LSMs) with overall standard errors (SE).

## Results

### Daily diet intake

**Figure 1:**
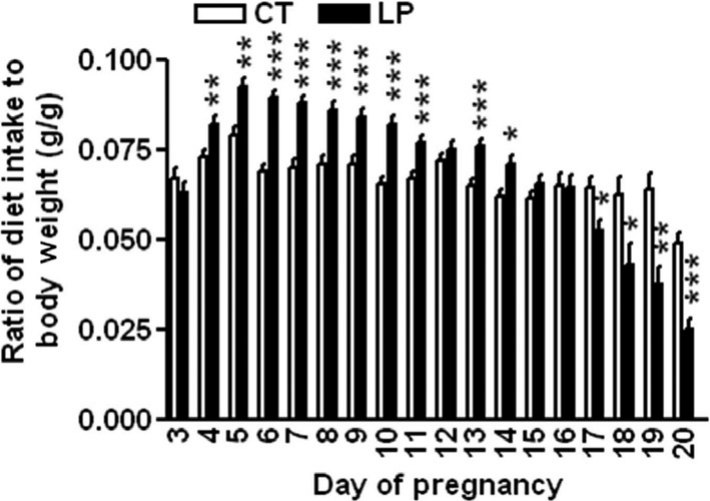
Daily diet intake. CT: control; LP, low-protein diet. The bar represents the mean ± SEM expressed as the ratio of diet intake (gram) to body weight (gram) of pregnant rats on each day of pregnancy (*n* = 8–10). *P <* *0.05; **P <* *0.01; ***P <* *0.001.

During Days 4–11 and 13–14 of pregnancy, the ratio of daily diet intake to body weight was 4.3–29.8% higher in the LP group than in the CT group (*P *<* *0.05). In contrast, during Days 17–20 of gestation, the ratio of daily diet intake to body weight was 18.2–48.9% lower in the LP group than in the CT group ((*P *<* *0.05; Fig.1).

### Maternal body weight changes

**Figure 2:**
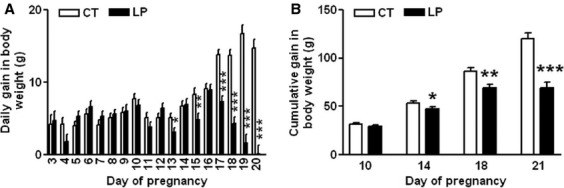
Daily gain in body weight during Day 3–20 of gestation (A) and cumulative gain in body weight on Days 10, 14, 18, and 21 of gestation (B). CT: control; LP, low-protein diet. The bar represents the mean ± SEM (*n* = 8–10). *P <* *0.05; **P <* *0.01; ***P <* *0.001.

Daily gain in body weight was similar between LP and CT groups during gestational Days 3–12, and lower (*P *<* *0.05) in the LP group on Days 13, 15, 17–20. On Days 17–20 of gestation, the gain in body weight in LP rats was (*P *<* *0.001) reduced to 53.1%, 31.5%, 9.7%, and 0.7% of that in CT rats, respectively (Fig.2A). The cumulative gain in body weight was unchanged in LP rats on Day 10, but was reduced to 88.7% (*P *<* *0.05), 80.0% (*P *<* *0.01), and 55.4% (*P *<* *0.001) of that in CT rats on Days 14, 18, and 21 of gestation, respectively (Fig.2B).

### Expression of ghrelin production and activation-related genes in the stomach

**Figure 3:**
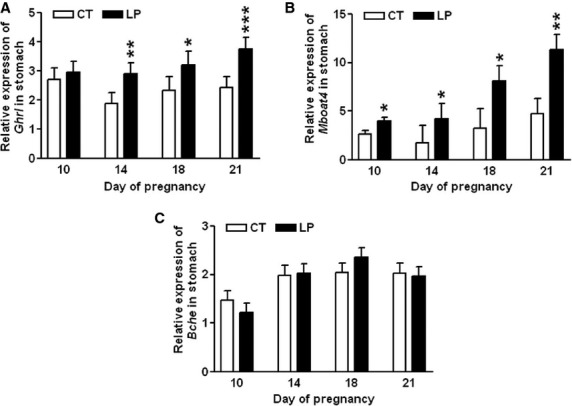
Quantitative real-time PCR analysis of *Ghrl* (A), *Mboat4* (B), and *Bche* (C) in the stomach on Days 10, 14, 18, and 21 of gestation. CT: control; LP, low-protein diet. The bar represents the mean ± SEM expressed as relative units of mRNA standardized against *Actb* (*n* = 8–10). *P <* *0.05; **P <* *0.01; ***P <* *0.001.

The mRNA levels of *Ghrl* in the stomach were unchanged in LP rats compared to CT rats on Day 10 of gestation, but were increased by 1.55-fold (*P *<* *0.01), 1.38-fold (*P *<* *0.05), and 1.55-fold (*P *<* *0.001) in LP rats on Days 14, 18, and 21 of gestation, respectively (Fig.3A). The mRNA levels of *Mboat4* in the stomach were also increased by 1.53-fold (*P *<* *0.05), 2.36-fold (*P *<* *0.05), 2.48-fold (*P *<* *0.05), and 2.40-fold (*P *<* *0.01) in LP rats on Days 10, 14, 18, and 21, respectively (Fig.3B). However, the mRNA levels of *Bche* in the stomach were unchanged in LP rats compared to CT rats on Days 10, 14, 18, and 21 of gestation (Fig.3C).

### Appetite regulation marker and ghrelin signaling gene expressions in the hypothalamus

**Figure 4:**
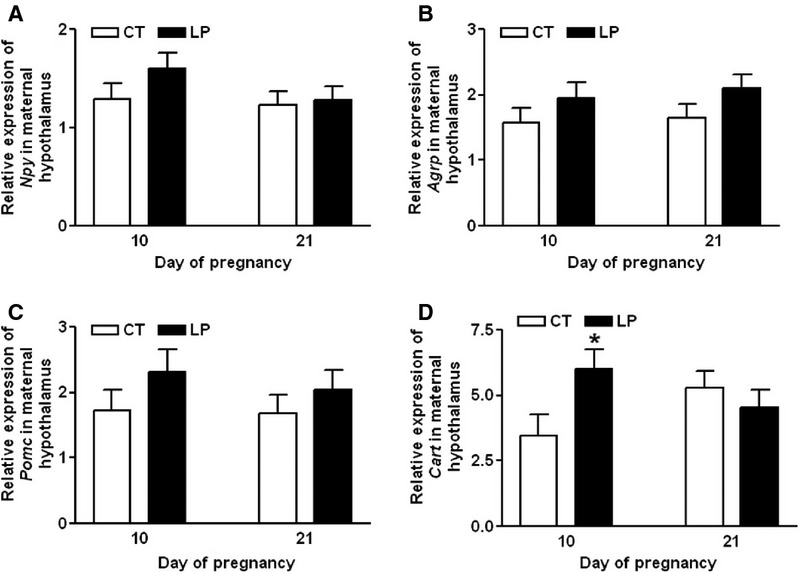
Quantitative real-time PCR analysis of *Npy* (A), *Agrp* (B), *Pomc* (C), and *Cart* (D) in the hypothalamus on Days 10 and 21 of gestation. CT: control; LP, low-protein diet. The bar represents the mean ± SEM expressed as relative units of mRNA standardized against *Actb* (*n* = 8–10). *P <* *0.05.

Expression of *Npy*, *Agrp,* and *Pomc* in the hypothalamus*,* was unaltered in LP rats on Day 10 of gestation compared to that in CT rats, whereas expression of *Cart* was increased (*P *<* *0.05) in LP rats. On Day 21, mRNA expression of *Npy*, *Agrp*, *Pomc,* and *Cart* was not affected in LP rats (Figs.4A–D).

**Figure 5:**
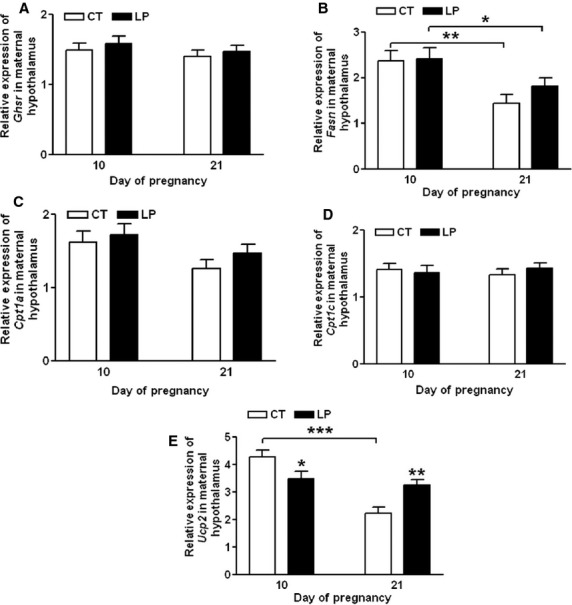
Quantitative real-time PCR analysis of *Ghsr* (A), *Fasn* (B), *Cpt1a* (C), *Cpt1c* (D), and *Ucp2* (E) in the hypothalamus on Days 10 and 21 of gestation. CT: control; LP, low-protein diet. The bar represents the mean ± SEM expressed as relative units of mRNA standardized against *Actb* (*n* = 8–10). *P <* *0.05; **P <* *0.01; ***P <* *0.001.

Expression of *Ghsr* (Fig.5A), *Fasn* (Fig.5B), *Cpt1a* (Fig.5C), and *Cpt1c* (Fig.5D) in the hypothalamus, was unaltered in LP rats on Days 10 and 21 of gestation compared to that in CT rats. Expression of *Fasn* in both CT and LP groups was reduced by 1.65-fold (*P *<* *0.01) and 1.32-fold (*P *<* *0.05), respectively, on Day 21 compared to Day 10 of gestation. Expression of *Ucp2* (Fig.5E) in LP group was decreased (*P *<* *0.05) on Day 10 and increased (*P *<* *0.01) on Day 21 of gestation; in CT rats, expression of *Ucp2* was decreased (*P *<* *0.05) by 1.92-fold on Day 21 compared to that on Day 10.

### Appetite regulation marker gene expressions in the ARC region of the hypothalamus

**Figure 6:**
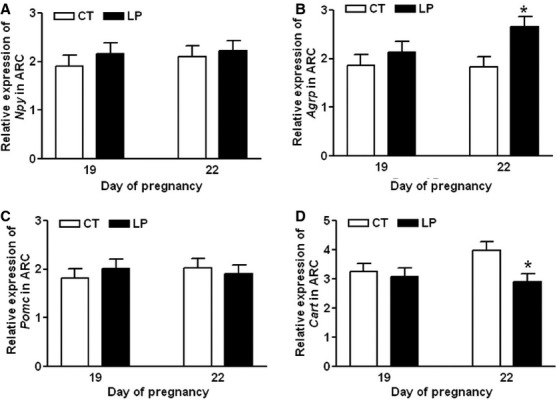
Quantitative real-time PCR analysis of *Npy* (A), *Agrp* (B), *Pomc* (C), and *Cart* (D) in arcuate nucleus region of the hypothalamus on Days 19 and 22 of gestation. CT: control; LP, low-protein diet; ARC, arcuate nucleus. The bar represents the mean ± SEM expressed as relative units of mRNA standardized against *Actb* (*n* = 8–10). *P <* *0.05.

Expression of *Npy*, *Agrp*, *Pomc,* and *Cart* in the ARC region of the hypothalamus was unaltered in LP rats compared to that in CT rats on Day 19 of gestation, whereas expression of *Agrp* was increased (*P *<* *0.05) and *Cart* was reduced (*P *<* *0.05) in LP rats on Day 22 (Fig.6A–D).

### Plasma levels of total and active ghrelin

**Figure 7:**
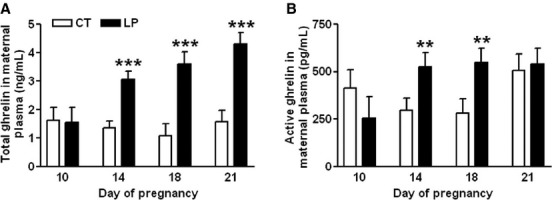
ELISA assays on plasma total (A) and active (B) ghrelin on Days 10, 14, 18, and 21 of gestation. CT: control; LP, low-protein diet; the error bar represents the mean ± SEM (*n* = 8–10). **P <* *0.01; ***P <* *0.001.

Total ghrelin levels in maternal plasma were similar in LP and CT rats on Day 10 of gestation, but were elevated (*P *<* *0.001) by 2.25-fold, 3.33-fold, and 2.74-fold in LP rats on Days 14, 18, and 21 of gestation, respectively (Fig.7A). The active ghrelin levels in maternal plasma were similar in LP and CT rats on Day 10 and 21, but were elevated (*P *<* *0.01) by 1.73-fold, and 1.96-fold i n LP rats compared to CT rats on Days 14 and 18, respectively (Fig.7B).

### Plasma levels of growth hormone

**Figure 8:**
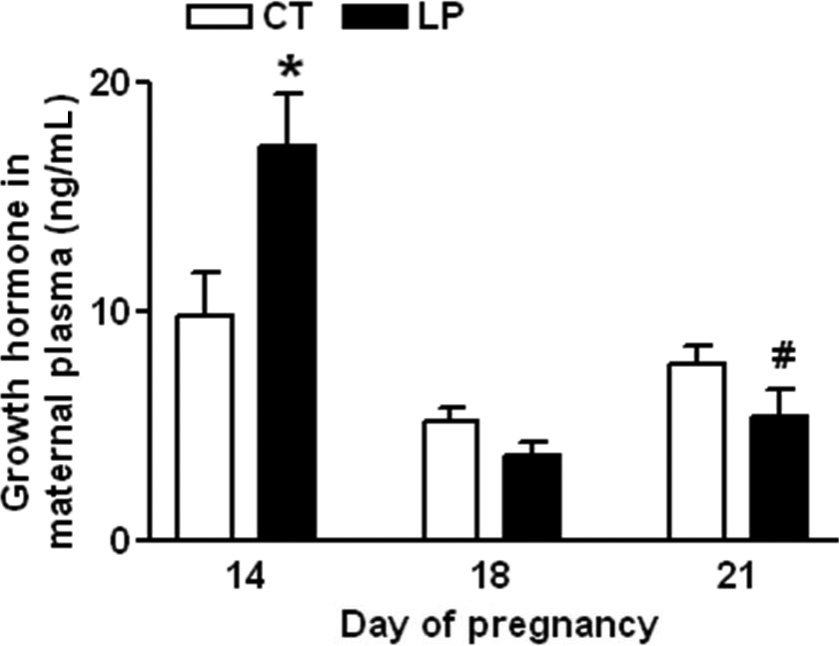
ELISA assay on plasma growth hormone on Days 14, 18, and 21 of gestation. CT: control; LP, low-protein diet. The bar represents the mean ± SEM (*n* = 8–10). *P <* *0.05; ^#^*P *=* *0.08.

Besides regulating the food intake, ghrelin signaling in the hypothalamus has been shown to alter the release of growth hormone from the pituitary (Sun et al. 2004; Kojima and Kangawa 2005). Because the majority of circulating growth hormone in pregnant rats is of pituitary origin (Carlsson et al. 1990), we investigated whether growth hormone levels are affected in LP rats. Growth hormone levels in maternal plasma in the LP group compared to CT group were increased (*P *<* *0.05) by 1.76-fold on Day 14, unchanged on Day 18, and slightly decreased (*P *=* *0.08) on Day 21 of gestation (Fig.8).

### Plasma levels of leptin

**Figure 9:**
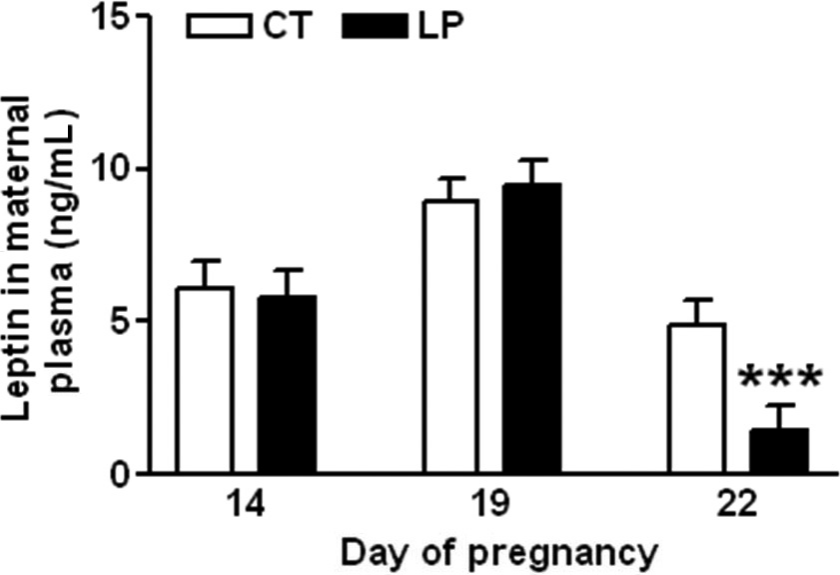
ELISA assay on plasma leptin on Days 14, 19, and 22 of gestation. CT: control; LP, low-protein diet. The bar represents the mean ± SEM (*n* = 8–10). ***P <* *0.001.

Lepin is a potent anorexigenic factor, thus it can offset the stimulatory effect of ghrelin in regulating food intake (Tena-Sempere 2007). We investigated whether changes in plasma leptin levels contribute to the reduced diet intake in LP rats in late pregnancy. Plasma leptin levels were similar in LP and CT rats on Days 14 and 19, but decreased (*P *<* *0.001) by threefold in the LP group compared to the CT group on Day 22 of gestation (Fig.9).

## Discussion

This study reports, for the first time, that a low-protein diet affects the diet consumption in pregnant rats. However, the variations in diet intake during pregnancy in the LP group are not associated with changes in the only known peripheral appetite stimulatory hormone, ghrelin. In particular, the elevated plasma ghrelin levels in LP rats were not associated with increased diet intake in late pregnancy, and therefore protein insufficiency in LP rats was exacerbated in late pregnancy. Thus, perhaps it is the enhanced catabolism of nutrient reservoir in LP pregnant dams, demonstrated by reduced body weight gain in this study (Fig.2B), which contributes largely to nutritional homeostasis in late pregnancy in both pregnant dams (Gao et al. 2012a) and fetuses (Rees et al. 1999). Findings in this study provide new information on the diet intake pattern in pregnant rats fed a low-protein diet and that diet intake is variable throughout gestation.

The diet intake in LP rats was biphasic, with increased intake during early pregnancy and reduced intake during mid- and late pregnancy (Fig.1). This differs from a previous report (Desai et al. 1996), in which similar diet consumptions were observed in both CT and LP groups. The discrepancy may be due to the differences in measurement methods; in the previous report (Desai et al. 1996) diet intake was expressed as cumulative weekly intake, whereas we measured daily intake, and thus, our study describes the dynamic changes in diet intake and body weight gain in pregnancy more rigorously in a species with relatively short gestation period.

Protein metabolism in the normal rat pregnancy includes anabolic and catabolic phases (Naismith and Morgan 1976), with Day 14 of pregnancy being the demarcation between these two phases (Naismith 1969). In the first 2 weeks of pregnancy, the ‘anabolic phase’, pregnant dams build up a reserve of nutrients, which are mobilized in the catabolic phase, to support the rapid growth of the feto-placental unit in late pregnancy (Naismith and Morgan 1976). In our study, the diet intake in LP rats was higher than in the control group during Days 3–12. The protein content is 20% in the CT diet and 6% in the LP diet. The increased diet intake in LP rats may partly compensate for the reduced amount of protein in the diet, but cannot make up for the significantly lower levels of protein in the LP diet. Thus, protein insufficiency could be a major factor in this rat model fed a low-protein diet in early and mid-pregnancy. In late pregnancy, the diet intake in LP rats was only 18.2–48.9% of that in CT rats during Day 17–20 of gestation, and thus the insufficiencies in both protein and energy could be exacerbated in LP rats. As a consequence, the gain in body weight was markedly reduced in late pregnancy (Fig.2B), and more of the nutrients accumulated during early pregnancy may be mobilized during late pregnancy. More importantly, the reduced diet intake in late pregnancy in LP rats results in decreases in other components of the diet. Among those diet components, taurine (Scabora et al. 2015) and folic acid (Torrens et al. 2006) supplemented to LP dams have been shown to rescue the adult offspring from developing hypertension. Added sucrose to the diet has been shown to influence the food intake, however, we suggest that the modest differences in the sucrose content of the LP vs. CT diet (57 vs. 43%), would not fully account for the differences in their diet intake. Thus, our study suggests the possible involvement of energy, macro- and micronutrients besides protein insufficiency in the process of fetal programming in LP rats.

Both total and active ghrelin are produced via complicated posttranslational processing (Kojima and Kangawa 2005), the positive correlation between the enhanced gene expression of *Ghrl* and *Mboat4* in the stomach and elevated plasma ghrelin levels has been reported in both normal-cycling and pregnant rats (Gualillo et al. 2002; Gonzalez et al. 2008; Reimer et al. 2010). In this study, enhanced expression of *Ghrl* in the stomach in LP rats was found during mid-late pregnancy (Fig.3), thus total ghrelin production in the stomach was also elevated in LP rats in mid-late pregnancy, and may contribute to the increased plasma levels of total ghrelin (Fig.7A). In contrast, the placenta may not significantly contribute to the increased levels of plasma ghrelin, because the expression of *Ghrl* is low and restricted to the placental labyrinth zone, and primarily occurs during late gestation (Gualillo et al. 2001). Moreover, expression of *Ghrl* in rat placenta was unchanged in LP rats (data not shown). Similar to total ghrelin, active ghrelin levels in plasma were increased in LP rats during mid-late pregnancy (Fig7B), coincident with the upregulation of *Mboat4* expression in the stomach (Fig.3B). Ghrelin-induced appetite stimulation has been shown by many paradigms (Kojima and Kangawa 2005; Naslund and Hellstrom 2007; Sobrino et al. 2014), thus, the elevated plasma levels of both total and active ghrelin may be a response to the insufficiency of both energy and nutrients (Langley-Evans et al. 1999). It is noteworthy that, on Day 21 of pregnancy, active ghrelin levels in the LP rats were similar to those in CT rats (Fig.7B), although the expression of *Mboat4* in the stomach was higher in LP rats than in CT rats (Fig.3B). This inconsistency may reflect reduced MBOAT4 activity in LP rats near term, which already experienced a prolonged fasting status, a situation similar to chronic undernutrition (Liu et al. 2008).

This study shows that changes in plasma levels of ghrelin were not associated with diet intake in pregnant rats fed a low-protein diet in either early or late pregnancy. During early pregnancy, when LP rats consumed a relatively larger amount of diet, the formation of active ghrelin (Fig.3B) as well as the expression of ghrelin-regulated genes in the hypothalamus (Fig.5) was not enhanced. This indicates that factors other than ghrelin may regulate appetite in early pregnancy by increasing expression of *Cart* (Fig.4D) and reducing expression of *Ucp2* in the hypothalamus (Fig.5E). It is known that ghrelin acts as a short-term appetite stimulator before diet intake (Kojima and Kangawa 2005), but after diet intake its orexigenic effect is quenched by many other hormones including insulin, glucagon-like protein 1, peptide YY, cholecystokinin, and oxyntomodulin (Naslund and Hellstrom 2007; Suzuki et al. 2011). In contrast, when LP rats consumed much lower amount of diet in late gestation (Fig.1), appetite-related gene expression in the hypothalamus (Figs.5) as well as in the ARC (Fig.6) was not increased, despite elevated total (Fig.7A) and active (Fig.7B) ghrelin levels in plasma. Therefore, some of the steps in ghrelin signaling in the hypothalamus may be blunted in the LP rats. It is noteworthy that on Day 22, the increased expression of *Agrp* (Fig.6B) and the decreased expression of *Cart* (Fig.6D) were coincident in the ARC of the LP rats, which were not found in the hypothalamus on Day 21 (Fig.4). The reduced expression of *Cart* may be due to the prolonged fasting-like status (Li et al. 2002) and together with the increased expression of *Agrp*, may represent an adaptation to long-term fasting. To explore whether the ghrelin insensitivity in food intake regulation is specific, we investigated the effect of the LP dieton plasma levels of growth hormone in pregnant rats, as the other known physiological function of ghrelin in the central nervous system is to stimulate growth hormone release from pituitary (Kojima et al. 1999) and pituitary is the primary source of circulating growth hormone in rats even during pregnancy (Carlsson et al. 1990). Plasma levels of growth hormone were not elevated on Day 18 and 21 of pregnancy (Fig.8) when plasma ghrelin levels were increased in LP rats (Fig.7). This suggests that not only ghrelin signaling for appetite stimulation, but also the ghrelin-induced growth hormone release may be blunted in LP rats It is noteworthy that although bolus i.v. injection of ghrelin to pregnant rats transiently elevates plasma levels of growth hormone (El-Kasti et al. 2008), the effect of sustained high levels of endogenous ghrelin on growth hormone remains unknown.

The stimulatory effect of ghrelin in diet intake in late pregnancy may be inhibited by other factors in LP rats. In this study, we ruled out interference by leptin on ghrelin-induced diet intake in LP rats. Leptin is a potent anorexigenic hormone, and acts as a regulator of appetite, energy homeostasis and reproduction (Tena-Sempere 2007). Plasma levels of leptin were not associated with the changes in diet intake in LP rats in late pregnancy, and indeed were decreased in LP rats when the diet intake was also substantially lower (Fig.9). Similarly, in a study using 4% protein diet fed pregnant rats, plasma leptin levels were also reduced (Jansson et al. 2006), which may upregulate circulating ghrelin levels (Barazzoni et al. 2003). Expressions of other anorexigenic hormones such as *Glp1*, *Glp2*, *Pyy*, *Ccka,* and *Cckb* in the small intestine were all unchanged in LP rats in late pregnancy (data not shown). Moreover, angiotensin II is known to inhibit appetite and ghrelin signaling pathway in the hypothalamus (Yoshida et al. 2012). Thus, significantly elevated angiotensin II levels during late pregnancy in LP rats (Gao et al. 2012b, 2013), could play a role in reducing diet intake. Therefore, the inhibitory effects of angiotensin II may offset the stimulatory effects of ghrelin on diet intake in LP rats.

In this study, we found that in pregnant rats fed a low-protein diet, changes in food intake over the course of pregnancy were not correlated with changes in ghrelin. During early pregnancy the production of total ghrelin and formation of active ghrelin in the stomach are not affected by the LP diet. During mid- and late pregnancy, the production of total ghrelin and formation of active ghrelin in the stomach are elevated by the LP diet. However, despite this, diet intake in LP rats is not increased during late pregnancy and is insufficient to support the rapid fetal growth.
